# IFN-α_2b_ reduces released particles of Human T-lymphotropic Virus-I from HTLV-I transformed cell line

**DOI:** 10.1186/1742-4690-8-S2-P30

**Published:** 2011-10-03

**Authors:** Christine Hartoonian, Mahsa Rasekhian, Rozita Edalat, Arash Arashkia, Kayhan Azadmanesh

**Affiliations:** 1 Department of Pharmaceutical Biotechnology, Faculty of Pharmacy, Tehran University of Medical Sciences, Tehran, Iran; 2 Virology department, Pasteur Institute of Iran, Tehran, Iran

## Background

HTLV-I is causative agent of Acute T-cell Leukemia (ATL) and HTLV-I associated myelopathy/tropical spastic paraparesis (HAM/TSP). To date there isn't an established treatment available for HTLV-I infection. Type-I IFNs are well known for their antiviral, anti proliferative and immunomodulatory effects. Antiviral mechanism of these IFNs varies depending on the virus and host type and could fluctuate between virus entry to its replication and release. IFN-α _2b_ is qualified for treatment of Hepatitis B and C and some malignancies such as specific leukemias and lymphomas. There are few clinical trials indicating type-l IFNs efficacy in HTLV-I infected patients,[1] nevertheless a few studies have focused on antiviral mechanism of alpha IFNs on HTLV-I.[2,3] Here we report the reducing effect of IFN-α _2b_ (PDferon^®^-B) on released particles of HTLV-I by HTLV-I transformed MT2 cell line.

## Materials and methods

MT2 cells were treated with 1000 U/ml of IFN-α _2b_ ( PDferon^®^-B). 12 and 36 hours post treatment, MT2 cells were collected and cell lysates were analyzed for expression of HTLV-I Gag, Tax and HBZ genes by gene specific primers utilizing real time RT-PCR method .As a representative of HTLV-I free particles released in supernate by MT2 cells, virus Gag gene copy number in supernate was evaluated at aforementioned hours post treatment.

## Results

There was no significant difference in expression of HTLV-I Gag, Tax and HBZ genes between cell lysates of treated and untreated MT2 cells at both 12 and 36 hours post treatment. At 12 hours post treatment, released Virus titer was not changed in supernate of treated cells while a significant decrease in viral particles was observed in collected supernate of treated cells at 36 hours after treatment with IFN-α_2b_.

## Conclusion

Our result shows that IFN-α_2b_ (PDferon^®^-B) treatment does not have any effect on expression of both structural and non structural genes of HTLV-I at 12 and 36 hours of treatment thus Reduction of HTLV-I titer at the 36^th^ hour post treatment suggests that IFN-α_2b_ ( PDferon^®^-B) does not exert its antiviral effects at viral transcription level. The significant decrease in viral titer in supernate by IFN-α _2b_ ( PDferon^®^-B) could be due to its interfering effect at any posttranscriptional level. Our results are in accordance with previous studies that have shown the similar effects of other members of type-l IFN family such as IFNα_2a_. [2]The exact mechanism by which this IFN has exerted its effects is yet to be clarified by details. Outcome of such extended studies would be of paramount importance in using IFN-α _2b_ in treatment of HTLV-I infection.
